# Metabolomics coupled with integrated approaches reveal the therapeutic effects of higenamine combined with [6]-gingerol on doxorubicin-induced chronic heart failure in rats

**DOI:** 10.1186/s13020-020-00403-0

**Published:** 2020-11-17

**Authors:** Jianxia Wen, Xiao Ma, Ming Niu, Junjie Hao, Ying Huang, Ruilin Wang, Ruisheng Li, Jian Wang, Yanling Zhao

**Affiliations:** 1 Department of Pharmacy, Chinese PLA General Hospital, Beijing, China; 2 College of Pharmacy, Chengdu University of Traditional Chinese Medicine, Chengdu, China; 3 Integrative Medical Center, Chinese PLA General Hospital, Beijing, China; 4 College of Pharmaceutical Science, Yunnan University of Chinese Medicine, Kunming, China; 5 Department of Traditional Chinese Medicine, Chinese PLA General Hospital, Beijing, China; 6 Research Center for Clinical and Translational Medicine, Chinese PLA General Hospital, Beijing, China

**Keywords:** Higenamine, [6]-gingerol, Metabolomics, Chronic heart failure, Energy metabolism, Molecular mechanisms

## Abstract

**Background:**

This study was aimed to investigate the therapeutic effects and potential mechanism of higenamine combined with [6]-gingerol (HG/[6]-GR) against doxorubicin (DOX)—induced chronic heart failure (CHF) in rats.

**Materials and methods:**

Therapeutic effects of HG/[6]-GR on hemodynamics indices, serum biochemical indicators, histopathology and TUNEL staining of rats were assessed. Moreover, a UHPLC-Q-TOF/MS-based serum metabolic approach was performed to identify the metabolites and possible pathways of HG/[6]-GR on DOX-induced CHF.

**Results:**

HG/[6]-GR had effects on regulating hemodynamic indices, alleviating serum biochemical indicators, improving the pathological characteristics of heart tissue and reducing the apoptosis of myocardial cells. Serum metabolisms analyses indicated that the therapeutic effects of HG and [6]-GR were mainly associated with the regulation of eight metabolites, including acetylphosphate, 3-Carboxy-1-hydroxypropylthiamine diphosphate, coenzyme A, palmitic acid, PE(O-18:1(1Z)/20:4(5Z,8Z,11Z,14Z)), oleic acid, lysoPC(18:1(9Z)), and PC(16:0/16:0). Pathway analysis showed that HG/[6]-GR on CHF treatment was related to twelve pathways, including glycerophospholipid metabolism, fatty acid metabolism, pantothenate and CoA biosynthesis, citrate cycle (TCA cycle), pyruvate metabolism, and arachidonic acid metabolism. Serum metabolites and metabolic pathways regulated by HG/[6]-GR appear to be related to energy metabolism.

**Conclusion:**

Multivariate statistical analysis has provided new insights for understanding CHF and investigating the therapeutic effects and mechanisms of HG/[6]-GR, which influencing the metabolites and pathways related to energy metabolism pathway.

## Background

Cardiovascular disease (CVD) causes a huge health and economic burden worldwide. According to the data from National Health and Nutrition Examination Survey from 2013 to 2016, the prevalence of CVD in adults ≥ 20 years is 48.0% in total, and the prevalence is increasing with advancing age in both males and females [1]. Chronic heart failure (CHF) is the terminal stage of various heart diseases. It remains a major clinical cause of morbidity, mortality and seriously endangers the health of humans globally [2]. It is a common cause of death with high direct and indirect treatment costs [3]. Currently, the problems experienced by patients and the medical community are extraordinary mortality, repeated hospitalization and combined therapies. The different kinds of pharmacological agents used for patients with CHF include angiotensin-converting enzyme inhibitors (ACEI), aldosterone antagonists, angiotensin-receptor blockers, aldosterone antagonists, β-blockers, inotropic agents, diuretics, digitalis, nitrates, vasodilators and so on [4, 5]. Although these agents are expected to be very major treatments, its poor prognosis and few therapeutic options make CHF still a growing global public health concern. The prevention and treatment of CHF remain major issues globally [6].

Natural medicine has the characteristics of the various component and complex mechanism, which has enormous potential in multiple disease treatment. Simultaneously, these products can act at multiple targets and pathways in the complex pathogenesis of diseases. In the prevention and treatment of CHF, traditional Chinese medicine (TCM) plays a distinct advantage with its multi-components, multi-target and multi-channel. The combination of Aconiti Lateralis Radix Praeparata (ALRP) and Zingiberis Rhizoma (ZR) is one of the most typical representatives reflecting the very essence of the theory of Chinese material media compatibility, which has been applied to treat cardiovascular disease for many years. Previously, we demonstrated that the ALRP-ZR prevented doxorubicin (DOX)-induced CHF in vivo. However, its functional components are remaining unclear. Our previous studies have shown that the compatibility use of higenamine (HG, one of the active compounds of ALRP) and 6-gingerol ([6]-GR, one of the active compounds of ZR) inhibits DOX-induced CHF via promoting mitochondrial energy metabolism [6]. However, the potential mechanism of HG/[6]-GR for the treatment of CHF has not been comprehensively elucidated. It remains to be elucidated how HG/[6]-GR can prevent and treat CHF by affecting mitochondrial energy metabolism.

Metabolomic is a comprehensive and systematic study of small molecule metabolites in biological samples or organs [7]. It can characterize changes of endogenous metabolites and their organic relations with physiological and pathological phenotypes after disturbance [8]. The metabolism of an organism changes its dynamic balance due to the occurrence of disease, so it is helpful to understand the metabolic mechanism of the organism by analyzing the composition of body fluid through metabolomics and obtaining biomarkers changed by disease induction. One of the basic methods of metabolomics research is the combination of advanced modern analytical technology, pattern recognition and expert system [9]. In recent years, metabolomics have also been used to identify specific biomarkers and evaluate the role of TCM in various diseases [10–12]. Therefore, the objective of current study was to use serum metabolomics analysis accompanied by biochemical and histopathological approaches investigating and verify the metabolic profiles of blood metabolite spectrum caused by the development of CHF, as well as the treatment of HG/[6]-GR. Simultaneously, this study was expected to reveal the anti-CHF mechanism of HG/[6]-GR in rats.

## Materials and methods

### Materials

Standard products of HG (CAS No.: 5843–65-2; Cat No. CHB180121, Degree of impurity: HPLC ≥ 98%) and [6]-GR (CAS No.: 23513–14-6; Cat No. CHB180306, Degree of impurity: HPLC ≥ 98%) were obtained from Chroma Biotechnology Co. Ltd (Chengdu, China). DOX hydrochloride injection was purchased from Shenzhen Main Luck pharmaceutical Inc. (batch number: 1710E1, Shenzhen, China). Dobutamine hydrochloride (DH) injection (Batch number: 1803203, Shanghai, China) was purchased from SPH NO.1 Biochemical & Pharmaceutical CO., LTD.

### Animal handling

Male Sprague–Dawley (SD) rats (200 ± 10 g) aged 6–8 weeks were purchased from Beijing Keyu Animal Breeding Center (Beijing, China) with a permission number of SCXK-(jing) 2018–0010. Rats were fed in the Animal Experiment Center of Chinese PLA General Hospital. Rats in the control group were intragastrically given normal saline. Simultaneously, rats in the other groups were given DOX hydrochloride injection in doses of 2.5 mg/kg body weight twice a week for six times. Thus, accumulative doses of DOX were 15 mg/kg body weight [13–15]. Hemodynamic indices were comprehensively assessed by a RM6240 multi-channel physiological signal acquisition system (Chengdu Instrument Factory, Sichuan, China) [16–19]. When the values of + dp/dt_max_ were reduced to 50% of the control group, CHF model was successfully prepared.

Rats with successfully prepared CHF model were randomly assigned into five groups of eight rats in each group: DOX group, DH positive group (50 μg/kg/d), HG group (5 mg/kg/d), [6]-GR group (5 mg/kg/d), and HG/ [6]-GR compatibility group (10 mg/kg/d). Eight rats in the control group received the same volume of normal saline. All rats were intraperitoneally injected with corresponding drugs once a day for seven consecutive days. It should be noted that CHF rats intraperitoneally injected with 5 mg/kg/d HG and [6]-GR showed a beneficial therapeutic effect in our previous study [20]. Hemodynamic indices were assessed after the final injection. All animals were sacrificed to collect serum samples and cardiac tissues for pharmacodynamic and serum metabolomic analysis.

### Detection of pharmacodynamic indices

Serum biochemical indices, including BNP, NT-proBNP, LDH, CK-MB, and AST were determined on a Synergy hybrid reader (Biotek, Winooski, USA). In addition, the serum energy metabolism-related indices, including ATP, ATPase, NAD, NADH were also detected. BNP, NT-proBNP, LDH, and CK-MB were recruited from Shanghai MLBIO Biotechnology Co., Ltd. AST was obtained from Nanjing Jiancheng Bioengineering Institute. ATP, ATPase, NAD, and NADH were purchased from Shanghai Kanglang Biotech Co., Ltd. Terminal deoxynucleotidyl transferase dUTP nick end labeling (TUNEL) assay was performed to indicate the cytotoxicity, cell damage and its recovery.

### Preparation of serum metabolomics samples

Firstly, the serum samples of rats were thawing at 4 °C conditions. 200 μL of serum was mixed with 600 μL of methanol to precipitate the protein. After centrifugation at 13,800 *g*, 4 °C for 10 min, the supernatant was transferred into a polypropylene tube and filtered via a syringe filter (0.22 μm) for obtaining the injection sample. Simultaneously, to assess the stability and reproducibility of serum metabolomics samples, the quality control (QC) sample was prepared by mixing all individual samples with 10 μL aliquots each.

### Chromatography analysis

The serum samples were measured on an Agilent 1290 series UHPLC system (Agilent Technologies, Santa Clara, USA) coupled with a ZORBAX RRHD 300 SB-C18 column (100 × 2.1 mm, 1.8-μm, Agilent Technologies, Santa Clara, USA) for chromatography and separation. During the analysis, the setting conditions were set as follows: sample maintaining temperature, 4 °C; injection volume: 4 μL; column temperature: 30 °C; flow rate, 0.30 mL/min. The mobile phases were composed as solvent A (0.1% formic acid in acetonitrile), and solvent B (0.1% formic acid in water). The gradient elution was setting as Table 1.

**Table 1:** Mobile phases for serum metabolomics analysis

T (min)	A (v/v)%	B (v/v)%
0–1.0	95	5
1.0–9.0	95–60	5–40
9.0–19.0	60–10	40–90
19.0–21.0	10–0	90–100
21.0–25.0	0	100

A, 0.1% formic acid in acetonitrile; B, 0.1% formic acid in water

### Mass spectrometry analysis

Mass spectrometry analysis was performed using an Agilent 6550A Q-TOF/MS instrument (Agilent Technologies, Santa Clara, USA) coupled with an electrospray ionization (ESI) source in both positive and negative ionization mode in the full scan mode (80–1200 m/z). The setting conditions in mass spectrometry analysis were as follows: gas temperature: 225 °C in positive ionization mode and 200 °C in negative ionization mode; nozzle voltage: 500 V in both positive and negative mode; electrospray capillary voltage, 4.0 kV in positive ionization mode and 3.0 kV in negative ionization mode; nebulizer: 45 pisg (positive) and 35 pisg (negative); gas flow rare: 11 L/min; mass range: from 80 to 1000 m/z; sheath gas flow: 12 L/min; sheath gas temperature: 350 °C.

### Data processing and multivariate data analysis

After statistical analysis by MetaboAnalyst 4.0 (https://www.MetaboAnalyst.ca/) [21], the raw data were converted into “data_normalized.csv” format. Then, the normalized file in positive mode and negative mode were imported into the SIMCA-P program (version 14.1, MKS Umetrics) for multivariate analysis, respectively. Principal component analysis (PCA) was performed after concentration and normalization to check the overall metabolism of each sample group, and observe sample aggregation, dispersion and abnormal values. Next, orthogonal partial least-squares discriminant analysis (OPLS-DA) was used to identify the main difference variables that caused the aggregation and discretization. Subsequently, 100 iteration permutation tests were performed to avoid the over-fitting of OPLS-DA. Potential biomarkers were selected according to the parameters of variable VIP > 1 and |Pcorr|> 0.58 from OPLS-DA. SPSS 23.0 software with the *t*-test was used to test the peak areas of differential metabolites and determine the differences of biomarkers between groups (p-value threshold was set at 0.05).

### Potential metabolites identification and pathway analysis

Furthermore, a MassHunter Profinder software (version B.06.00, Agilent, California, USA) was utilized to analyze the sample data for peak detection and alignment. Full scans mode was employed and the mass range was 80–1000 m/z. The biochemical online database HMDB database (https://www.hmdb.ca/) and METLIN (https://metlin.scripps.edu/) were used to identify the potential metabolites. MetaboAnalyst 4.0 was used for the pathway analysis. Finally, to identify and visualize the affected metabolic pathways, the biomarkers were put into MetaboAnalyst 4.0 based on the pathway library of Rattus norvegicus (rat). In the present study, the bioactive components, possible biomarkers and potential mechanisms of HG and [6]-GR in the treatment of CHF induced by DOX were comprehensively elucidated using the serum metabolomics strategy.

### Statistical analysis

All data were analyzed using SPSS 23.0 software programmes (Chicago, United States) and GraphPad Prism 8.2.0 software (GraphPad Software). The differences of data between groups were assessed by one-way analysis of variance (ANOVA). Values in the text were presented as mean ± SD. *P* < 0.05 was considered statistically significant. *P* < 0.01 was considered highly significant.

## Results

### Hemodynamics indices

The therapeutic effects of HG/[6]-GR on heart function were evaluated by assessing the hemodynamics indices. Compared with the control group, DOX could substantially decrease the LVSP and + dp/dt_max_ value while significantly increase the LVEDP and -dp/dt_max_ value, indicating that the model of CHF was successfully prepared. However, compared with the DOX group, DH, HG, [6]-GR, and HG/[6]-GR could dramatically increase the levels of LVSP and + dp/dt_max_ and decrease the LVEDP and -dp/dt_max_ value. The order of the therapeutic effects was HG/[6]-GR > HG > [6]-GR. Also, compared with HG and [6]-GR used alone, HG/[6]-GR group had a more superior effect on increasing heart function (Table 2).

**Table 2:** Effects of HG/[6]-GR on hemodynamic indices in rats

Group	LVSP (mmHg)	LVEDP (mmHg)	+ dp/dt_max_ (mmHg/s)	-dp/dt_max_ (mmHg/s)
C	140.56 ± 12.75	− 12.97 ± 2.85	7506.87 ± 668.49	− 5330.51 ± 884.92
DOX	51.57 ± 8.38^**^	10.54 ± 1.83^**^	2313.95 ± 416.24^**^	− 1386.08 ± 172.61^**^
DH	132.04 ± 10.48^##^	− 7.40 ± 0.89^##^	8579.56 ± 873.29^##^	− 6757.02 ± 601.40^##^
HG	112.30 ± 10.91^##^	− 1.47 ± 2.59^##^	9034.06 ± 426.66^##^	− 6778.09 ± 291.51^##^
[6]-GR	100.91 ± 12.02^##^	7.34 ± 0.86^##^	5029.14 ± 256.68^##^	− 3729.03 ± 104.06^##^
HG/[6]-GR	125.25 ± 7.92^##abb^	− 4.07 ± 0.93^##abb^	10,263.24 ± 1056.67^##aabb^	− 8428.85 ± 399.13^##aabb^

Compared with the control group

**P < 0.01; compared with the DOX group

^##^*P* < 0.01; compared with the HG group

^a^*P* < 0.05

^aa^*P* < 0.01; compared with the [6]-GR group

^bb^*P* < 0.01

### Myocardial biomarkers

**Fig. 1:**
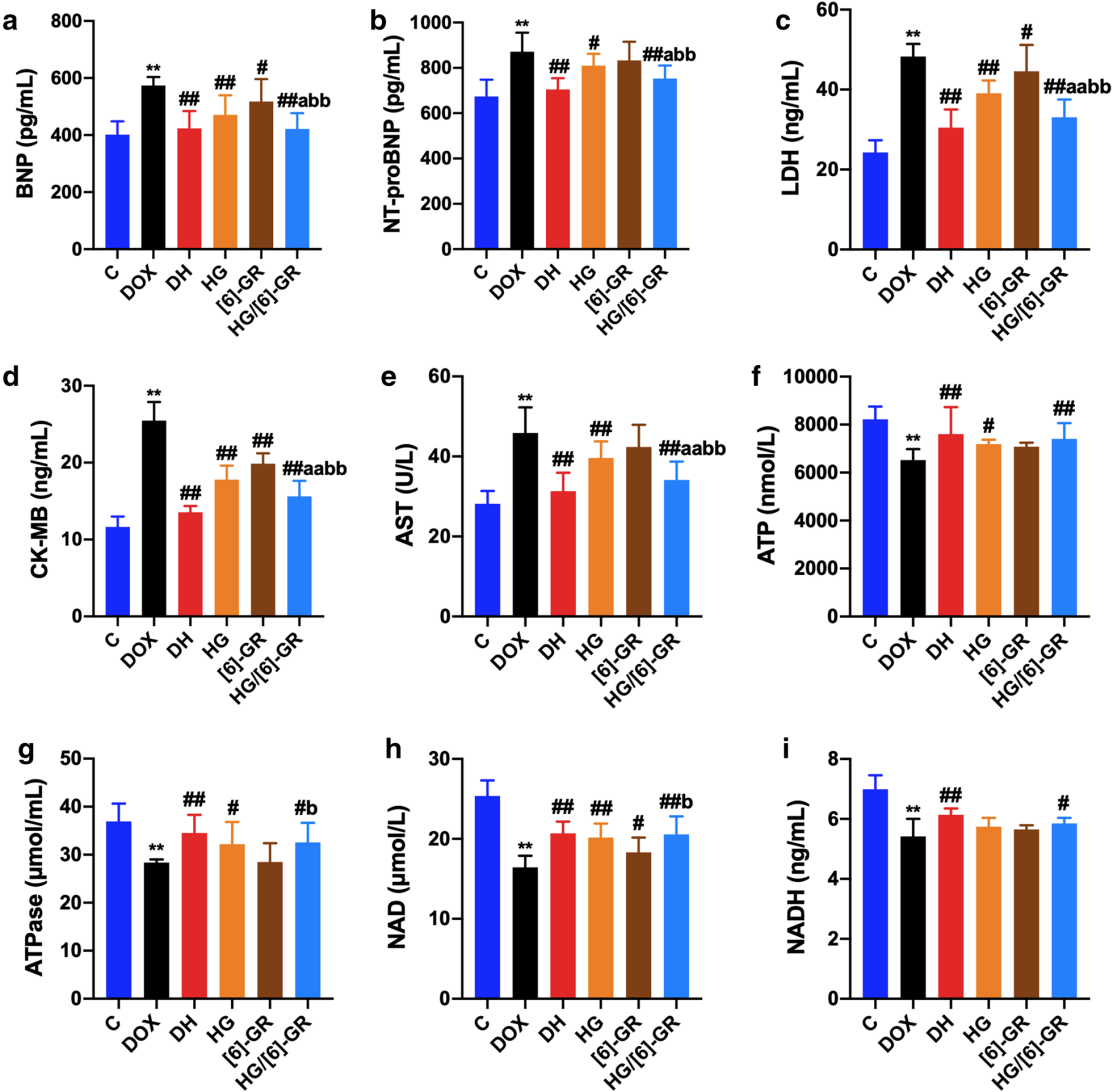
The serum levels of the myocardial biomarkers and energy metabolism indices for the six groups. **a** BNP; **b** NT-proBNP; **c** LDH; **d** CK-MB; **e** AST, **f** ATP; **g** ATPase; **h** NAD; (I) NADH. Compared with the control group, **P < 0.01; compared with the DOX group, ^#^*P* < 0.05, ^##^*P* < 0.01; compared with the HG group, ^a^*P* < 0.05, ^aa^*P* < 0.01; compared with the [6]-GR group, ^b^*P* < 0.05, ^bb^*P* < 0.01

Serum levels of myocardial biomarkers were included in Fig. 1. Compared with the control group, serum levels of BNP, NT-proBNP, LDH, CK-MB, and AST in the DOX group were significantly increased (*P* < 0.01) while serum levels of ATP, ATPase, NAD, and NADH were decreased in the DOX group (*P* < 0.01), which indicated the damage of heart function and energy metabolism disorder in DOX group. However, compared with the DOX group, HG and [6]-GR could reduce the serum concentrations of BNP, NT-proBNP, LDH, CK-MB, and AST, but increase the serum levels of ATP, ATPase, NAD, and NADH. Notably, these biomarkers were substantially changed in DH and HG/[6]-GR (*P* < 0.01) group compared with the DOX group. Furthermore, HG/[6]-GR group was almost equal to the DH group, which markedly decreased the serum levels of BNP, NT-proBNP, LDH, CK-MB, and AST but increased the levels of ATP, ATPase, NAD, and NADH compare with HG or [6]-GR used alone (*P* < 0.05, *P* < 0.01). Thus, [6]-GR might enhance the therapeutic role of HG in the treatment of CHF.

### Histopathological changes

**Fig. 2:**
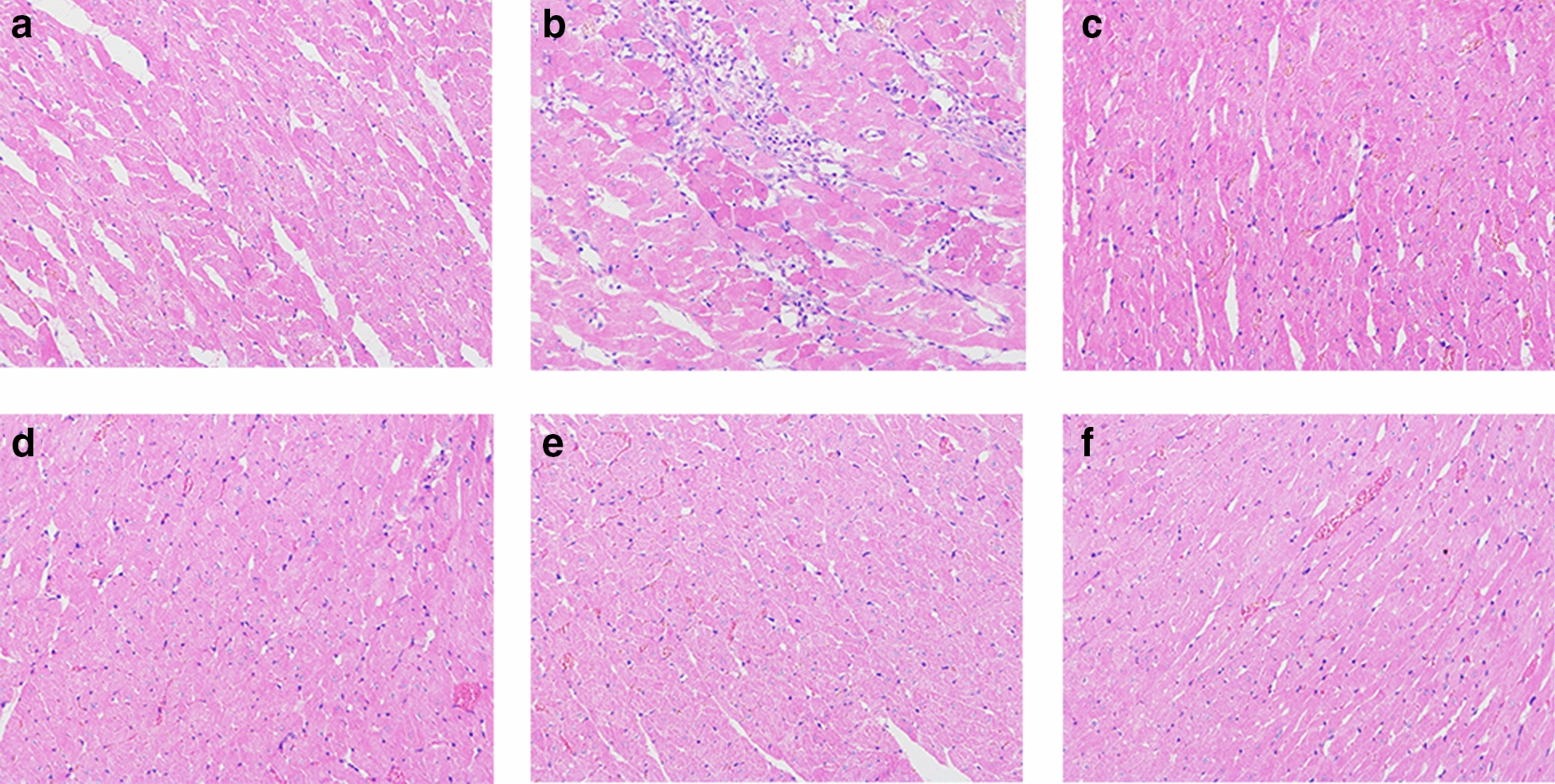
Effects of HG/[6]-GR on pathological changes of left ventricle in CHF rats (HE staining, 200 ×). **a** Control group; **b** DOX group; **c** DH group; **d** HG group; **e** [6]-GR group; **f** HG/[6]-GR group

The histopathological results showed the degree of damage in each group. After administration, the rats in DOX group had pathological changes such as widening and breaking of myocardial tissue space, vacuolar degeneration, edema, and necrosis of myocardial cells (Fig. 2b). Compared with the DOX group, the histopathology of HG and [6]-GR group was improved, but some rats still had widened and broken myocardial tissue space, vacuolation and degeneration of myocardial cells (Fig. 2d, e), while DH and HG/[6]-GR group showed significant improvement in cardiac pathology, less vacuolation, edema, necrosis, atrophy and other pathological changes of myocardial cells (Fig. 2c, f).

### Detection of cardiomyocyte apoptosis

**Fig. 3:**
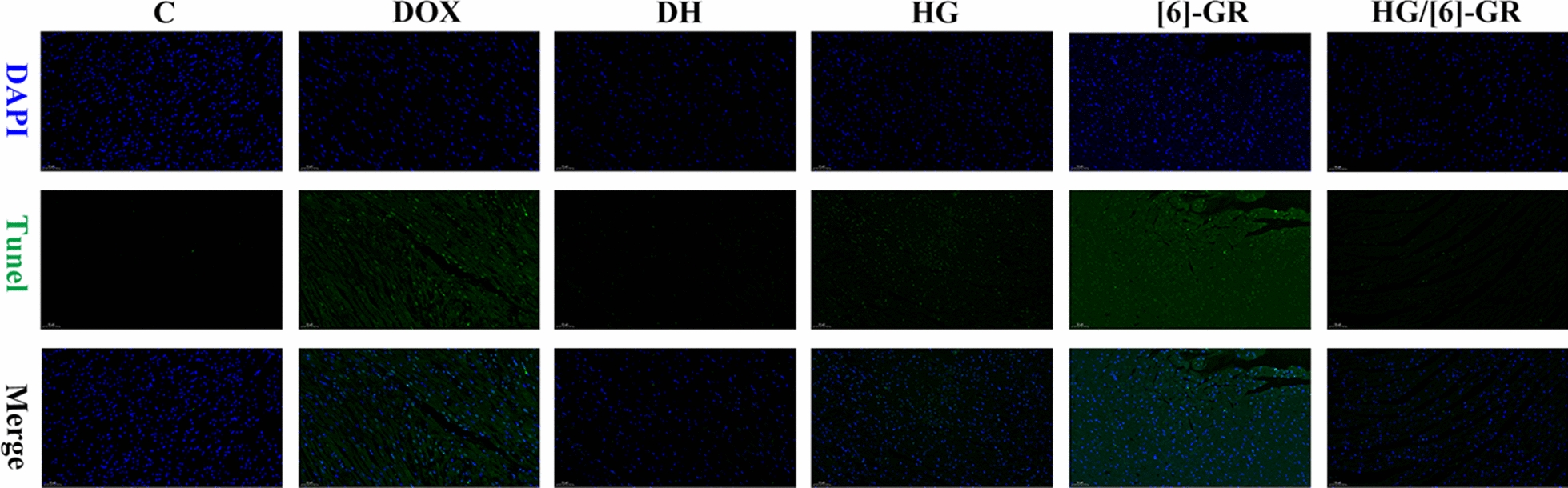
Effects of HG/[6]-GR on cardiomyocyte apoptosis of left ventricle in CHF rats (Tunel staining, 200 ×)

TUNEL staining was used to detect the therapeutic effect of HG/[6]-GR on DOX induced cardiomyocyte apoptosis and its recovery. As shown in Fig. 3, compared with the control group, the TUNEL positive proportion of cardiomyocytes in DOX treatment group increased significantly, indicating that DOX could cause cardiomyocyte apoptosis. In contrast, HG and [6]-GR used alone could reduce the apoptosis rate of cardiomyocytes in varying degrees. Moreover, HG/[6]-GR had a significant inhibitory effect on cardiomyocyte apoptosis, indicating that HG combined with [6]-GR had a synergistic anti-apoptotic effects. These results showed that HG/[6]-GR had a significant protective effect on CHF myocardial tissue.

### Metabolic profile analysis

**Fig. 4:**
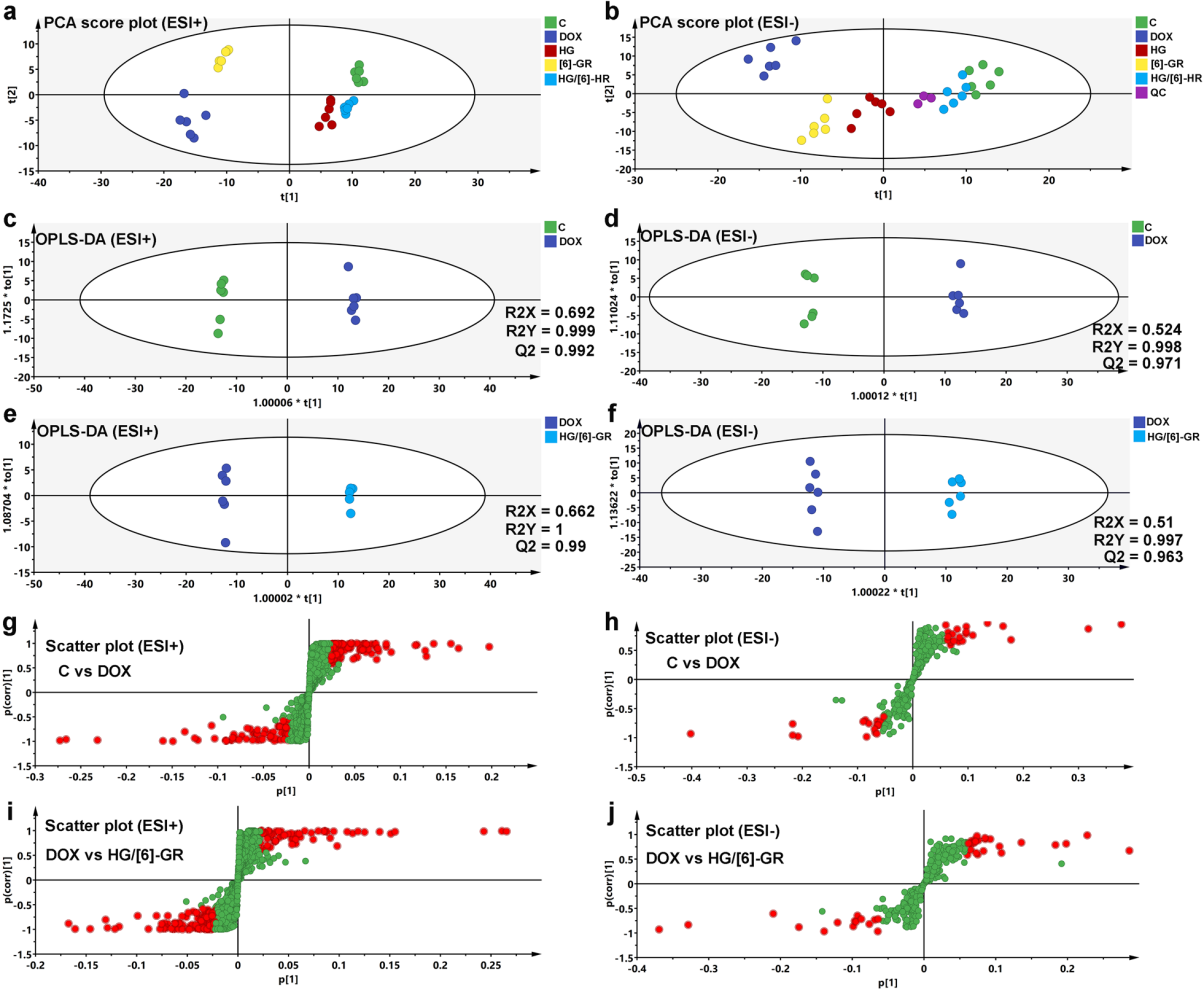
PCA and OPLS-DA score plots of the serum samples from the control, DOX, and HG/[6]-GR groups. PCA score plots in the positive mode (**a**) and negative mode (**b**); OPLS-DA score plots of control and DOX groups in the positive mode (**c**) and negative mode (**d**); OPLS-DA score plots of DOX and HG/[6]-GR groups in the positive mode (**e**) and negative mode (**f**); Scatter plot of control and DOX groups in the positive mode (**g**) and negative mode (**h**); Scatter plot of DOX and HG/[6]-GR groups in the positive mode (**i**) and negative mode (**j**)

**Fig. 5:**
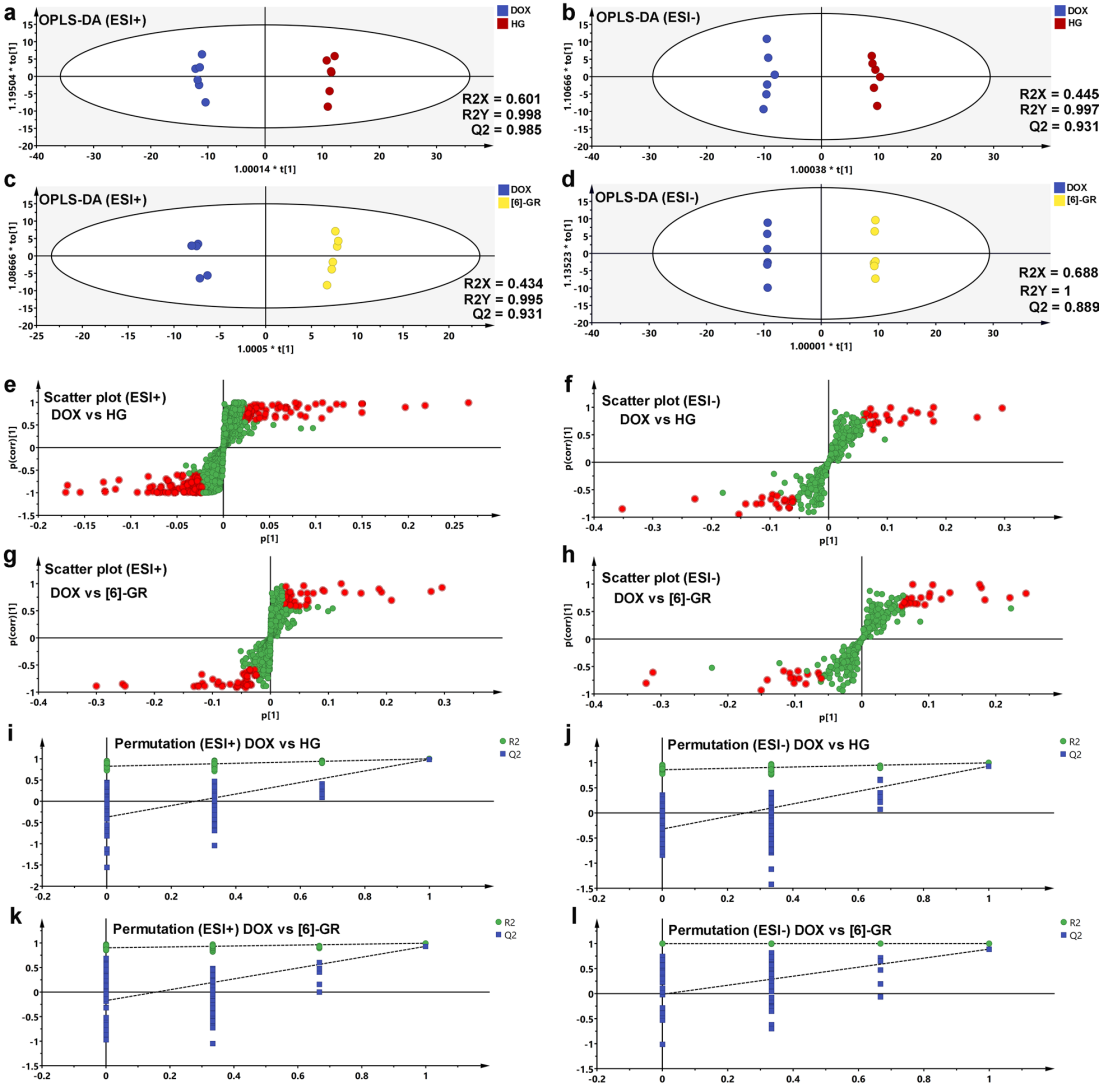
OPLS-DA score plot, scatter plot, and 100 iteration permutation of the serum samples among the DOX, HG and [6]-GR groups. OPLS-DA score plots of DOX and HG groups in the positive mode (**a**) and negative mode (**b**); OPLS-DA score plots of DOX and [6]-GR groups in the positive mode (**c**) and negative mode (**d**); Scatter plot of DOX and HG groups in the positive mode (**e**) and negative mode (**f**); Scatter plot of DOX and [6]-GR groups in the positive mode (**g**) and negative mode (**h**); 100 iteration permutation tests of DOX and HG groups in the positive mode (**i**) and negative mode (**j**); 100 iteration permutation tests of DOX and [6]-GR groups in the positive mode (**k**) and negative mode (**l**)

Metabolic profile analysis of serum samples was performed using UHPLC-Q-TOF/MS both in the positive and negative electrospray ionization (ESI) modes. PCA analysis was performed to assess alterations in the metabolism of each group. In the PCA score plot (Fig. 4a, b), the control groups and DOX groups were clearly divided into two clusters. In addition, the HG/[6]-GR and HG groups were significantly separated from DOX group and closer to the control group, especially the HG/[6]-GR group. Furthermore, to maximize the difference of metabolic profiles, OPLS-DA analysis was carried out subsequently (Fig. 4c, d). The results showed that the OPLS-DA models were verified by the class permutation and all these models had predictive ability with an R^2^Y (cum), and Q^2^ (cum). The corresponding value had been marked in the Fig. 4c–f. The OPLS-DA model was performed based on the control group and model group, the R^2^Y (cum) and Q^2^Y (cum) were 0.999 and 0.992 in ESI + mode, 0.998 and 0.971 in the ESI- mode, respectively. Also, the OPLS-DA model was performed based on the DOX and HG/[6]-GR group (Fig. 4e, f). The DOX group could be clearly separated from the HG/[6]-GR group. The R^2^Y (cum) and Q^2^Y (cum) were 1 and 0.99 in the ESI + mode, 0.997 and 0.963 in the ESI- mode, respectively. In addition, metabolic profile analysis between the DOX and HG or [6]-GR group in the positive mode and negative mode was also performed (Fig. 5a–d). Scatter plots of the control and DOX group, DOX and HG/[6]-GR group were shown in Fig. 5e–h, and 100 iteration permutation tests of the DOX and HG group, DOX and [6]-GR group were shown in Fig. 5i–l.

### Identification and quantification of potential biomarkers

**Fig. 6:**
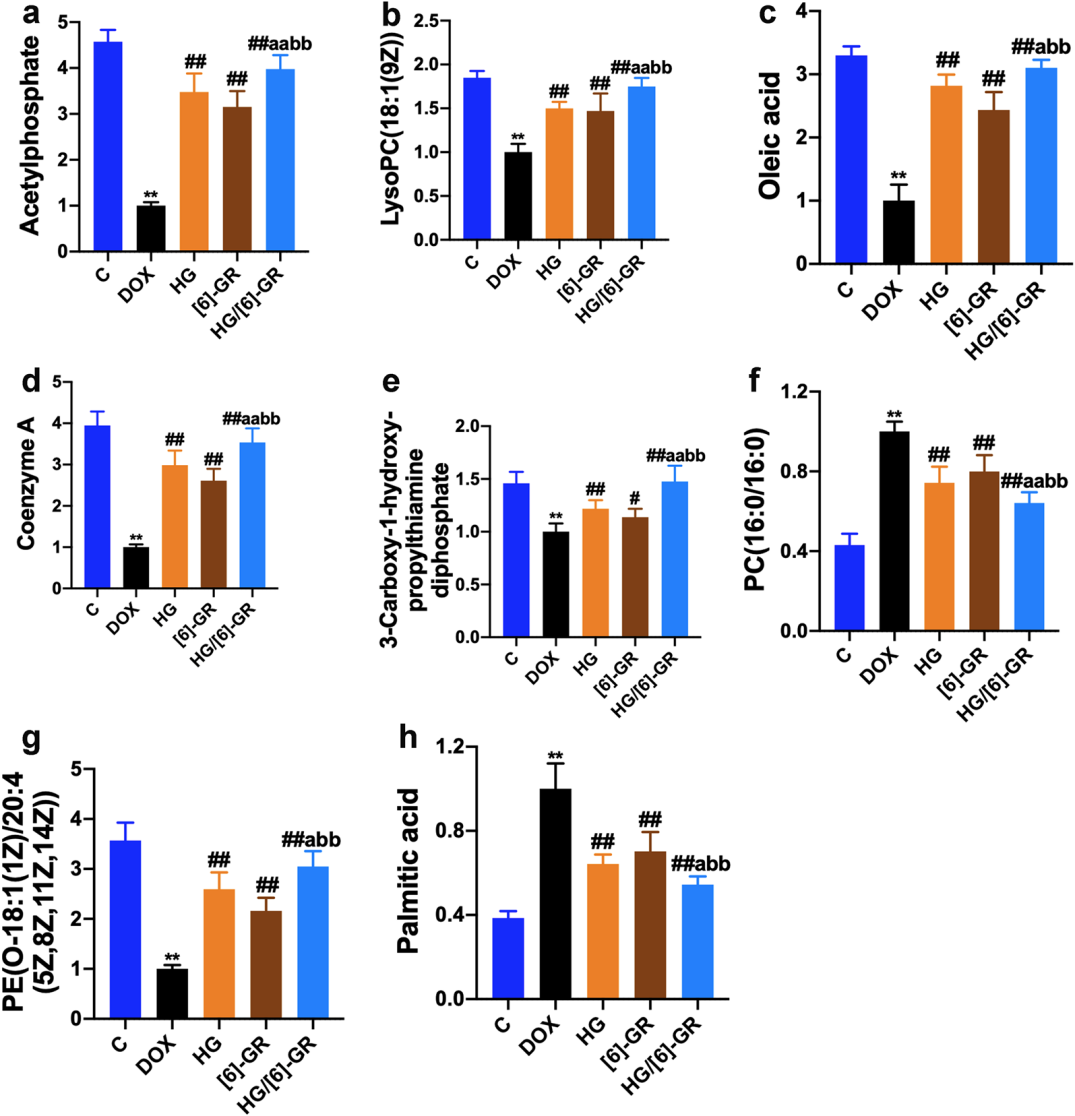
Potential biomarkers changes in DOX-induced CHF with HG/[6]-GR treatment. **a** Acetylphosphate; **b** LysoPC(18:1(9Z)); **c** Oleic acid; **d** Coenzyme A; **e** 3-Carboxy-1-hydroxypropylthiamine diphosphate; **f** PC(16:0/16:0); **g** PE(O-18:1(1Z)/20:4(5Z,8Z,11Z,14Z)); **h** Palmitic acid. Compared with the control group, **P < 0.01; compared with the DOX group, ^#^*P* < 0.05, ^##^*P* < 0.01; compared with the HG group, ^a^*P* < 0.05, ^aa^*P* < 0.01; compared with the [6]-GR group, ^bb^*P* < 0.01

Next, differential metabolites in CHF treatment were identified. The variables that substantially contributed to the clustering and identification were identified when their VIP values ≥ 1.0 and |p(corr)| values ≥ 0.58 in scatter plots. Finally, eight potential metabolites were expressed at significant levels and identified as biomarkers for the treatment of CHF. The basic characteristics of these potential biomarkers were summarized in Table 3 with their compound name, formula, mass (m/z), retention time (min), and ratio changes (significance). Next, the mechanism of action of HG/[6]-GR on DOX-induced CHF and the changes of eight possible metabolites were assessed and discussed. Compared with the control group, DOX substantially decreased peak area of acetylphosphate, 3-carboxy-1-hydroxypropylthiamine diphosphate, coenzyme A, PE(O-18:1(1Z)/20:4(5Z,8Z,11Z,14Z)), oleic acid, anslysoPC(18:1(9Z)) (Fig. 6a–e, g), but increase the peak area of PC(16:0/16:0) (Fig. 6f) and palmitic acid (Fig. 6h). Conversely, HG/[6]-GR could reverse these changes and decrease the peak area of PC(16:0/16:0) and palmitic acid. Notably, most of the metabolites indicated the formation of mitochondrial energy metabolism substrate. Overall, the results indicated that HG/[6]-GR had obvious therapeutic effects on DOX-induced CHF. Especially, the curative effect of HG/[6]-GR group was better than that of HG and [6]-GR used alone (Fig. 6).

**Table 3:** Identified metabolites of the serum sample from different groups

No	Compound Name	Formula	Mass (m/z)	Retention time (min)	Ratio changes (significance)	
					Control/DOX	HG/[6]-GR /DOX
1	Acetylphosphate	C_2_H_5_O5P	139.9872	20.01	4.57^**^	3.98^##^
2	3-Carboxy-1-hydroxypropylthiamine diphosphate	C_16_H_25_N_4_O_10_P_2_S	527.0692	13.79	1.46^**^	1.48^##^
3	Coenzyme A	C_21_H_36_N_7_O_16_P_3_S	767.1152	11.45	3.95^**^	3.54^##^
4	Palmitic acid	C_16_H_32_O_2_	256.2402	16.37	0.39^**^	0.54^##^
5	PE(O-18:1(1Z)/20:4(5Z,8Z,11Z,14Z))	C_37_H_66_NO_8_P	683.4375	20.46	3.57^**^	3.05^##^
6	Oleic acid	C_18_H_34_O_2_	287.2819	7.84	3.30^**^	3.10^##^
7	LysoPC(18:1(9Z))	C_24_H_51_NO_6_P	480.3086	15.87	1.85^**^	1.75^##^
8	PC(16:0/16:0)	C_46_H_83_NO_8_P	808.5856	15.33	0.43^**^	0.64^##^

Compared with the control group

**P < 0.01; compared with the DOX group

^##^*P* < 0.01

### Pathway analysis of CHF treatment

**Fig. 7:**
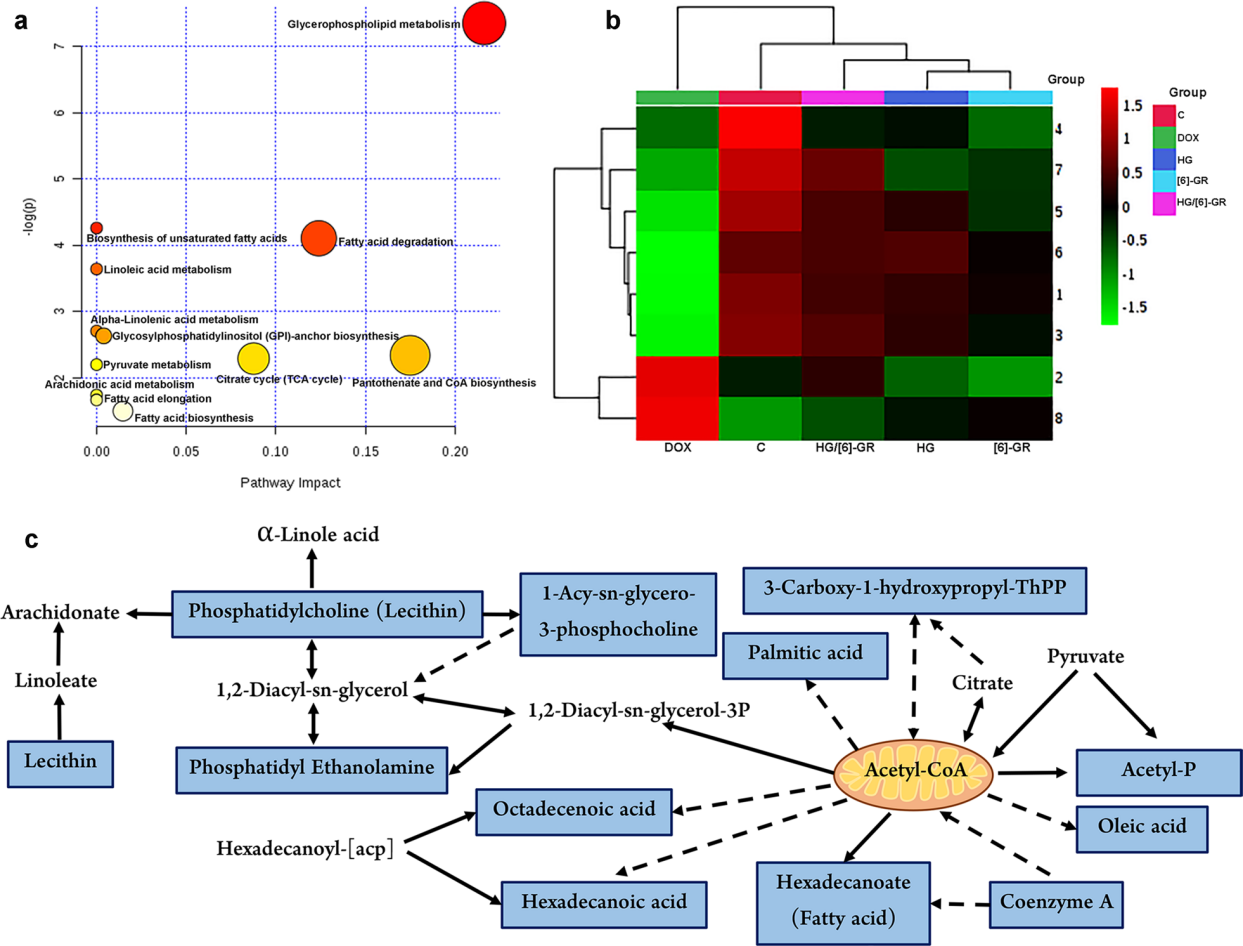
Metabolic biomarkers changes and related metabolic pathways involved in the treatment of HG/[6]-GR on CHF induced by DOX. **a** The metabolic pathways involved in the therapeutic effects of HG/[6]-GR on CHF; **b** The cluster heatmap of potential metabolites among groups. 1. Acetylphosphate; 2. 3-carboxy-1-hydroxypropylthiamine diphosphate; 3. coenzyme A; 4. palmitic acid; 5. PE(O-18:1(1Z)/20:4(5Z,8Z,11Z,14Z)); 6. oleic acid; 7. lysoPC(18:1(9Z)); 8. PC(16:0/16:0); **c** Relationship among the metabolic biomarkers and metabolic pathways

To explore the possible pathway of HG/[6]-GR and DOX intervention in CHF, the KEGG ID of endogenous metabolites was imported into the MetaboAnalyst 4.0 system for the pathway analysis and visualization. The results showed that CHF-related metabolites were responsible for energy metabolism pathway, including glycerophospholipid metabolism, biosynthesis of unsaturated fatty acids, fatty acid degradation, linoleic acid metabolism, alpha-Linolenic acid metabolism, glycosylphosphatidylinositol (GPI)-anchor biosynthesis, pantothenate and CoA biosynthesis, citrate cycle (TCA cycle), pyruvate metabolism, arachidonic acid metabolism, fatty acid elongation, and fatty acid biosynthesis (Fig. 7a). Besides, to determine the distribution and differences between groups, the clustering heat map was constructed based on the potential biomarker data (Fig. 7b). The match status, *p* value, -log(*p*) and the impact of each metabolic pathway were listed in Table 4. In addition, the relationship among metabolic pathways and metabolites was presented in the Fig. 7c. The recovery trend of metabolites showed that the therapeutic effect of HG/[6]-GR on heart was related to the above eight metabolic biomarkers and twelve metabolic pathways.

**Table 4:** Results of integrating pathway analysis with MetaboAnalyst 4.0

No	Pathway Name	Match status	*p*	− log(p)	Impact
1	Glycerophospholipid metabolism	3/36	0.00064401	7.3478	0.21631
2	Biosynthesis of unsaturated fatty acids	2/36	0.014161	4.2573	0
3	Fatty acid degradation	2/39	0.016523	4.103	0.12404
4	Linoleic acid metabolism	1/5	0.026263	3.6396	0
5	Alpha-Linolenic acid metabolism	1/13	0.067028	2.7026	0
6	Glycosylphosphatidylinositol (GPI)-anchor biosynthesis	1/14	0.072017	2.6308	0.00399
7	Pantothenate and CoA biosynthesis	1/19	0.096614	2.337	0.175
8	Citrate cycle (TCA cycle)	1/20	0.10146	2.288	0.08764
9	Pyruvate metabolism	1/22	0.1111	2.1973	0
10	Arachidonic acid metabolism	1/36	0.17603	1.7371	0
11	Fatty acid elongation	1/39	0.18939	1.6639	0
12	Fatty acid biosynthesis	1/47	0.2241	1.4957	0.01472

## Discussion

Cardiomyocyte energy metabolism, especially fatty acid and glucose metabolism, changes in CHF, and is considered to be a factor of heart function impairment in patients with HF [22]. Fatty acid β-oxidation is a process in which fatty acids decompose to produce ATP. In a series of steps of long-chain coenzyme A (COAs) entering mitochondria, COAs are converted into long-chain acyl coenzyme by carnitine palmitoyltransferase 1 (CPT1) [23]. Long chain acyl CoA can enter into β-oxidation of fatty acids. One acetyl CoA is generated from each cycle by this pathway as well as NADH and FADH2. The NADH and FADH2 produced by β-oxidation of fatty acids and the TCA cycling of the acetyl CoA are used by electron transport chain for producing ATP [24]. In addition, the metabolism of fatty acids is a major energy source under the conditions of hunger, starvation, infection and diabetic ketoacidosis. In the state of CHF, the mitochondrial fatty acids metabolism is significantly impaired. In return, inhibition of fatty acid metabolism can cause myocardial insufficiency [25, 26].

The pharmacodynamic effects of HG/[6]-GR on CHF were systematically evaluated. Firstly, the multi-channel physiological signal detection system was used to evaluate the CHF model. The results showed that + dp/dt_max_ value had been reduced to 50% of the control group, indicating the successful preparation of CHF model. Secondly, the system was used to detect the therapeutic effect of HG/[6]-GR on CHF. Surprisingly, HG/[6]-GR could significantly increase the + dp/dt_max_ value of CHF rats, and their combination was comparable to that of DH group. As serum BNP and NT-proBNP levels are the most widely used biomarkers in the diagnosis and treatment of HF, which are helpful for the diagnosis, differential diagnosis, risk stratification, efficacy monitoring and prognosis evaluation of acute-HF (AHF) and CHF [27]. Serum LDH, CK-MB, and AST levels can be used to evaluate whether the myocardium is damaged [28]. These parameters were comprehensively detected in the current study. The results showed that HG/[6]-GR could significantly reduce the increase of serum BNP, NT-proBNP, LDH, CK-MB, and AST caused by DOX. To characterize the therapeutic effect of HG/[6]-GR on myocardial energy metabolism in CHF rats, serum levels of ATP, ATPase, NAD, NADH were detected. The results showed that DOX significantly decreased serum levels of ATP, ATPase, NAD, and NADH, while HG combined with [6]-GR significantly increased these serum indices in CHF rats, indicating that HG/[6]-GR could reverse the damage of DOX on energy metabolism of rat cardiomyocytes. Overall, combined with the results of cardiac histopathology and TUNEL staining, HG/[6]-GR could improve the changes of myocardial histopathology and reduce the apoptosis of cardiomyocytes. According to the results of pharmacodynamic study, HG/[6]-GR might have a significant therapeutic effect on DOX-induced CHF.

Our previous study has shown that HG in combination with [6]-GR can substantially increase the CPT-1 level decreased by DOX, which can relieve cardiomyocyte injury induced by DOX via regulating fatty acid metabolism in the TCA cycle based on cell metabolomics [20]. In the present study, serum metabolomics coupled with integrative pharmacology has further improved our understanding of the therapy of DOX induced CHF with HG/[6]-GR from several pivotal aspects. Furthermore, an UHPLC-Q-TOF/MS-based serum metabolomics approach was used to study serum metabolites changes in CHF. Moreover, we demonstrated the therapeutic effects of HG/[6]-GR against CHF in rats, which specifically caused a significant restoration of their myocardial metabolic profiles. This alteration laid the foundation for further investigation into the fundamental mechanisms of HG/[6]-GR in the treatment of CHF. Eight metabolites were identified in the CHF treatment, including acetylphosphate, 3-carboxy-1-hydroxypropylthiamine diphosphate, coenzyme A, palmitic acid, PE(O-18:1(1Z)/20:4(5Z,8Z,11Z,14Z)), oleic acid, lysoPC(18:1(9Z)), and PC(16:0/16:0), which are distributed in twelve metabolic pathways. Most of the detected compounds are intermediates of energy metabolism. Among the changes of these potential metabolic pathways, the most obvious abnormality occurs in energy metabolism, which indicates that CHF is related to the disorder of energy metabolism in the heart. These findings are consistent with the previous studies [29–31]. Among these metabolites, acetylphosphate can phosphorylate biologically significant substrates in a way similar to ATP, promoting the origin of metabolism [32]. Coenzyme A is mainly involved in the metabolism of fatty acids and pyruvate, which can stimulate the tricarboxylic acid (TCA) cycle and provide 90% of the energy reqiured for the body's life [30]. Palmitic acid diets can cause lipo-toxicity and energy metabolism imbalance in vivo and in vitro [33]. Specifically, palmitic acid treatment can induce cardiomyocyte apoptosis, which is manifested by the appearance of apoptosis nucleus, the activation of caspase 3, the release of mitochondrial cytochrome C and the loss of mitochondrial cardiolipin [34]. Our results showed that DOX could substantially decrease the level of acetylphosphate and coenzyme A, but increase palmitic acid, indicating the damage to myocardial energy metabolism. Nevertheless, HG/[6]-GR could significantly reverse this change and affect the fatty acid metabolism and the citrate cycle. As fatty acid metabolism is a notable mechanism for creating energy for the heart and a significant target for storing or creating energy for the heart [35, 36], HG/[6]-GR may play a crucial role in the treatment of CHF by improving the energy metabolism function of myocardial mitochondria.

The comprehensive treatment has been advocated for thousands of years by TCM prescription, which is a special medical system to help ancient Chinese treat diseases. It is believed that multiple components of TCM can hit multiple targets and play a synergistic therapeutic effect [37]. Currently, researchers have performed various of studies have confirmed that Chinese medicine can improve the symptoms of CHF in different degrees, and elaborated its mechanism of action. The compatibility of ALRP and ZR is commonly used in clinical practice in ancient and modern times. A number of studies have confirmed the objective truth of ALRP combined with ZR in the treatment of CHF from the perspective of mitochondrial energy metabolism, but the material basis and mechanism of its regulation of myocardial energy metabolism are still unclear. A various studies have been done on the cardiotonic effect and potential mechanism of HG worldwide [38–40]. HG is a selective activator of beta2-adrenergic receptor, which plays a wide range of roles in blood vessels, bronchus and heart with positive inotropic effects [41, 42]. [6]-GR is a novel AT1 antagonist, which can regulate blood pressure and enhance the heart function in the cardiovascular system [43]. In this study, HG from ALRP combined with [6]-GR in ZR were used to investigate the effect of HG/[6]-GR on serum metabolic markers of DOX-induced CHF rats, and to explore its possible mechanism from the perspective of metabolomics. From the perspective of mitochondrial energy metabolism, the potential mechanism of HG, [6]-GR and their compatibility in the treatment of CHF is more conducive to further study of ALRP, ZR and their compatibility in improving the cardiac function.

In this study, although the effectiveness and potential mechanism of HG combined with [6]-GR in the treatment of CHF have been elucidated by a comprehensive method, some limitations still exists: (a) this study indicates that HG/[6]-GR might play a role in the treatment of CHF by affecting myocardial energy metabolism, the gene and protein expression of related pathways have not been verified to confirm the target mechanism of HG and [6]-GR; (b) the present study only discussed the effect of HG and [6]-GR on several biomarkers of metabolic difference, but the specific effect on other metabolites of tricarboxylic acid cycle remains to be elucidated; (c) in addition to myocardial energy metabolism, the causes of CHF include apoptosis and inflammation, whether HG and [6]-GR can play the role of CHF treatment through other channels remains to be studied; (d) the biomarkers of HG/[6]-GR affecting CHF have been discussed in serum metabolism level, how HG/[6]-GR affect these different biomarkers and what is the mode of action remains to be further studied. Therefore, although targeting mitochondrial energy metabolism is a promising strategy for the treatment of CHF, further studies are needed to confirm the potential beneficial effect of regulating these metabolic targets as a method for the treatment of CHF.

## Conclusions

The present study was to explore the therapeutic effect and the possible mechanism of HG/[6]-GR in the treatment of CHF specifically induced by DOX and increase understanding of CHF based on the serum metabolomics. Compared with the control group, the myocardial metabolic spectrum of CHF rats was significantly altered. Furthermore, the different metabolic markers of the control group, DOX group and HG/[6]-GR group were mainly involved in the cardiac energy metabolism. In addition, the cardiac function and histopathology of the HG/[6]-GR group were significantly ameliorated. The therapeutic effect of HG/[6]-GR might be attributed to its recovery of the disordered of mitochondrial energy metabolism pathway. These findings might provide novel insights for clarifying the potential mechanism of CHF and help to investigate the therapeutic effects and mechanism of HG/[6]-GR in the treatment of CHF.

## Data Availability

The data used to support the findings of this study are available from the corresponding author upon reasonable request.

## Abbreviations

ALRP: Aconiti Lateralis Radix Praeparata
ZR: Zingiberis Rhizoma
HG: Higenamine
[6]-GR: [6]-Gingerol
DOX: Doxorubicin
CHF: Chronic heart failure
CVD: Cardiovascular disease
ACEI: Angiotensin-converting enzyme inhibitors
TCM: Traditional Chinese medicine
DH: Dobutamine hydrochloride
± dp/dtmax: Left ventricular pressure max or min
LVEDP: Left ventricular enddiastolic pressure
LVSP: Left ventricular systolic pressure
ELISA: Enzyme linked immunosorbent assay
BNP: Brain natriuretic peptide
LDH: Lactate dehydrogenase
CK-MB: Creatine kinase-MB
AST: Aspartate aminotransferase
ATP: Adenosine triphosphate
NADH: Nicotinamide adenine dinucleotide
HE: Hematoxylin–eosin
TUNEL: Terminal deoxynucleotidyl transferase dUTP nick end labeling
ESI: Electrospray ionization
QC: Quality control
PCA: Principal component analysis
OPLS-DA: Orthogonal partial least-squares discriminant analysis
